# Proposal of Limosilactobacillus secundus sp. nov., Limosilactobacillus reuteri subsp. pararodentium subsp. nov., Limosilactobacillus reuteri subsp. peregrinus subsp. nov. and Limosilactobacillus reuteri subsp. simiae subsp. nov., isolated from the gastrointestinal tract of vertebrate hosts

**DOI:** 10.1099/ijsem.0.007099

**Published:** 2026-03-06

**Authors:** Xinyu Guo, Yi Yang, Justina Su Zhang, Peipei Zhang, Jens Walter, Michael G. Gänzle, Fuyong Li

**Affiliations:** 1 Ministry of Education Key Laboratory of Molecular Animal Nutrition, Zhejiang University, Hangzhou, 310058, PR China; 2 Department of Animal Science and Technology, College of Animal Sciences, Zhejiang University, Hangzhou, 310058, PR China; 3 Department of Agricultural, Food & Nutritional Science, University of Alberta, Edmonton, AB, T6G 2E1, Canada; 4 Department of Animal Sciences, Colorado State University, Fort Collins, CO, 80523-1171, USA; 5 APC Microbiome Ireland, School of Microbiology, and Department of Medicine, University College Cork, Cork, T12 YT20, Ireland

**Keywords:** evolution, host adaptation, *Limosilactobacillus reuteri*, subspecies

## Abstract

A core genome-based phylogenomic analysis of representative strains of the species *Limosilactobacillus reuteri* identified eight lineages that differ from the six previously described *L. reuteri* subspecies. Four of them are represented by isolates obtained from intestinal digesta or faeces of rodents and primates, including strains LR77^T^ and LR80 (lineage X), mlc3^T^ and LR92 (lineage VIII), LR51^T^ and LR88 (lineage VII) and LR66^T^ and LR52 (lineage IX). Analyses of pairwise average nucleotide identity and digital DNA–DNA hybridization values further support their genetic divergence from existing *L. reuteri* subspecies. These findings suggest the classification of these four lineages as one novel species of *Limosilactobacillus* and three new subspecies of *L. reuteri*. Therefore, we propose the novel species *Limosilactobacillus secundus* sp. nov. (type strain LR77^T^=DSM 113335^T^=LMG 32469^T^) and the novel subspecies *L. reuteri* subsp. *pararodentium* subsp. nov. (type strain mlc3^T^=DSM 113337^T^=LMG 32470^T^), *L. reuteri* subsp. *peregrinus* subsp. nov. (type strain LR51^T^=DSM 113336^T^=LMG 32467^T^) and *L. reuteri* subsp. *simiae* subsp. nov. (type strain LR66^T^=DSM 113334^T^=LMG 32468^T^). This expanded taxonomic framework enhances our understanding of the genetic diversity of *L. reuteri* and further supports its adaptations to diverse vertebrate hosts.

## Introduction

*Limosilactobacillus reuteri* serves as a model organism to study the ecology and evolution of host-associated lactobacilli. The ability of *L. reuteri* to thrive in diverse host species underscores its evolutionary versatility [1,2], which is further supported by the finding that this species has an open pangenome [3,4]. Analyses of the phylogeny of strains of *L. reuteri*, their gene content, their source of isolation and their ability to colonize mice identified ten lineages with distinct genomic, metabolic and ecological features [4]. Of these lineages, six are validly published as subspecies of *L. reuteri* [5]. The host specificity of strains of *L. reuteri* has been experimentally confirmed in birds and rodents. Strains of *L. reuteri* subsp. *kinnaridis* exhibit superior ecological fitness when compared to strains from other lineages [6]. Strains of *L. reuteri* subsp. *murium* and *L. reuteri* subsp. *rodentium* and strains of lineages VII, VIII, IX and X colonize mice, while strains of other subspecies do not [4,6]. In addition to rodents and birds, strains of *L. reuteri* were also isolated from humans, captive primates and domestic animals like herbivores and swine [4,7]. It remains unclear whether the presence of *L. reuteri* in these hosts represents long-term adaptation or only temporary persistence. For example, the presence of *L. reuteri* in humans likely results from zoonotic transmission from domestic animals such as poultry or herbivores [4]. In addition to the ten lineages/subspecies that were characterized by ecological and metabolic studies, four additional lineages are represented by genomes but not by isolates and originate from unknown hosts [8].

The six validly published subspecies of *L. reuteri* share an intra-subspecies average nucleotide identity (ANI) of more than 97% and an inter-subspecies ANI of 94–97%. An intra-subspecies ANI of more than 97% was also reported for the four lineages that are represented by isolates [4,5] and the four lineages that are not [8]. This communication aims to define four lineages that are represented by isolates as novel species or subspecies.

## Isolation and ecology

Phylogenetic analyses of 182 genomes of *L. reuteri* revealed ten lineages, including six lineages conforming to previously proposed subspecies and four novel lineages that cannot be assigned to the known subspecies of *L. reuteri* [4]. For the present study, we selected eight representative strains (i.e. LR77^T^, LR80, mlc3^T^, LR92, LR51^T^, LR88, LR66^T^ and LR52) from these four new lineages for further analyses. Strains LR77^T^ and LR80 were isolated from the jejunum of striped field mice (*Apodemus agrarius*) in the Vilnius area of Lithuania. Strain LR92 was isolated from a faecal sample of a Vancouver Island marmot (*Marmota vancouverensis*) at the Zoo in Calgary, Canada. Strains LR51^T^ and LR52 were isolated from faecal samples of black howler monkeys (*Alouatta caraya*), while strain LR66^T^ was isolated from a faecal sample of a patas monkey (*Erythrocebus patas*) at the San Francisco Zoo, California, USA. Strain LR88 was isolated from a faecal sample of a red-rumped agouti (*Dasyprocta leporina*) at the Valley Zoo in Edmonton, Canada, and strain mlc3^T^ was isolated from the faeces of a laboratory mouse (*Mus musculus*) (Table 1).

**Table 1.** Genomic characteristics, host origins and quality of genome assemblies of eight strains classified as one novel *Limosilactobacillus* species and three novel *L. reuteri* subspecies

	*L. secundus* sp. nov.		*L. reuteri* subsp. *pararodentium* subsp. nov.		*L. reuteri* subsp. *peregrinus* subsp. nov.		*L. reuteri* subsp. *simiae* subsp. nov.	
Strain name	LR77^T^	LR80	mlc3^T^	LR92	LR51^T^	LR88	LR66^T^	LR52
Source of isolation	Striped field mouse (*A. agrarius*)	Striped field mouse (*A. agrarius*)	Mouse (*M. musculus*)	Marmota (*M. vancouverensis*)	Black howler monkey (*A. caraya*)	Red-rumped agouti (*D. leporina*)	Common patas monkey (*E. patas*)	Black howler monkey (*A. caraya*)
GenBank ID	GCA_020784695.1	GCA_020784665.1	GCA_000179435.1	GCA_020784525.1	GCA_020785115.1	GCA_020784555.1	GCA_020784875.1	GCA_020785135.1
Genome size (bp)	2,014,409	2,130,452	2,018,627	2,087,278	2,099,280	2,190,901	2,162,028	2,199,526
Coverage (×)	>500	>500	20	>500	>500	>500	>500	>500
G+C content (mol%)	38.4	38.4	38.5	38.6	38.6	38.6	38.5	38.4
No. of contigs	46	64	126	139	118	94	122	80
N50 (bp)	124,921	133,090	53,518	60,880	108,559	121,258	80,397	133,800
Total genes	1,954	2,118	2,042	2,165	2,013	2,160	2,086	2,156
No. of CDS*	1,873	2,036	1,886	2,066	1,921	2,068	1,993	2,071

Data source: National Center for Biotechnology Information (NCBI) RefSeq database (accessed: 22 July 2025).

* CDS=coding sequence.

## Genomic analyses

**Fig. 1.**
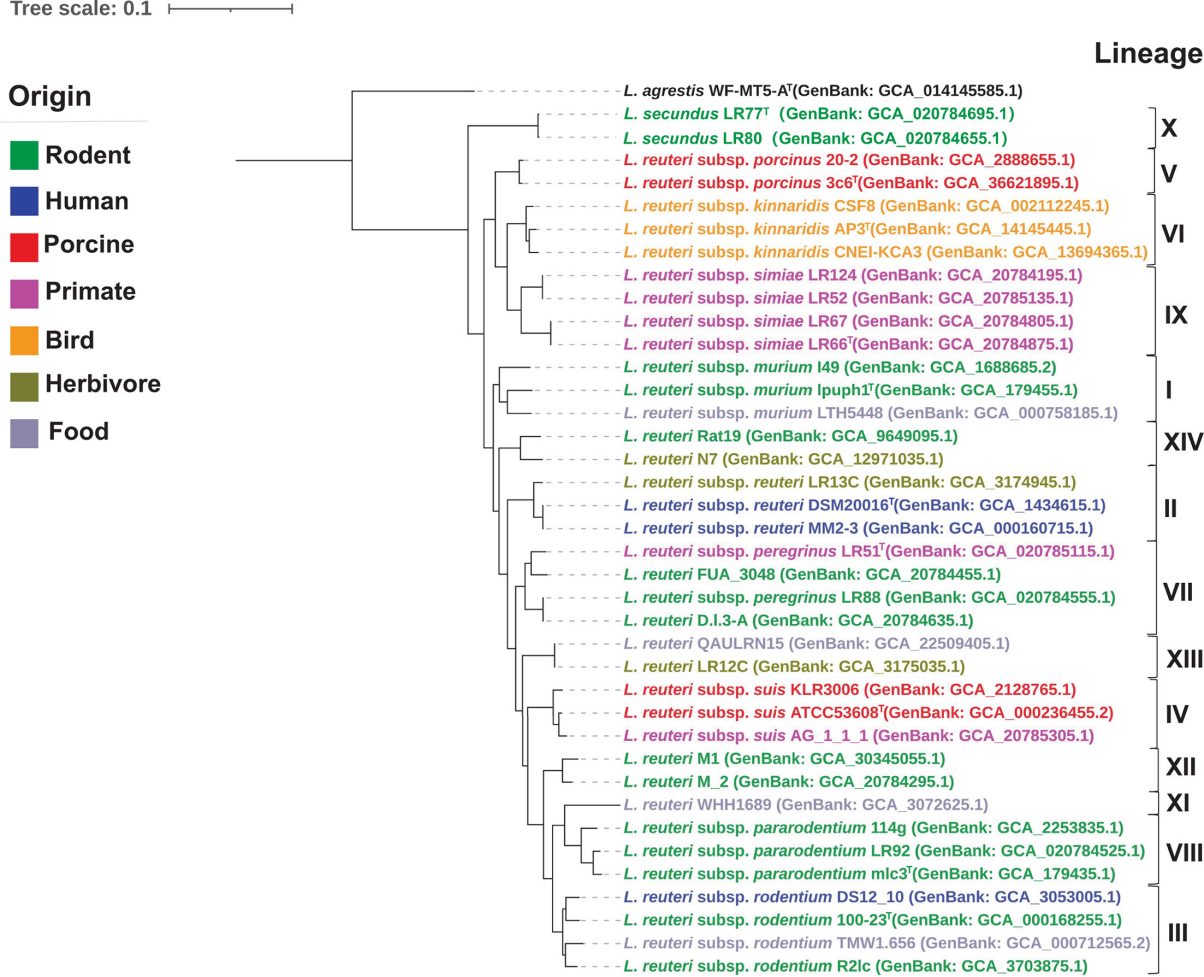
A maximum-likelihood phylogenetic tree was reconstructed using core genes identified from whole-genome sequences. The tree was inferred based on the GTR+G model with 1,000 bootstrap replicates. All branches in the phylogenetic tree exhibit higher than 80% bootstrap values. Genome sequences for type strains were obtained from the GenBank database. The 14 phylogenetic lineages of *L. reuteri* were numbered with Roman numerals to the right. The phylogenetic tree was visualized using iTOL [24].

The genomes of the eight strains of the four novel lineages were obtained from RefSeq database of the National Center for Biotechnology Information (NCBI) (Table 1). In addition, representative genomes of four lineages for which genomes became available since 2023 were included [8]. The size of genomes ranges from 2.01 to 2.20 Mbp, and G+C content ranges from 38.4 to 38.6 mol% (Table 1). To determine the phylogenetic position of the four novel lineages among *L. reuteri*, a phylogenetic tree was constructed using 38 strains of all known lineages of *L. reuteri*. The genome of *Limosilactobacillus agrestis* WF-MT5-A^T^ was used as an outgroup. All genomes were re-annotated using Prokka with default settings [9]. Core genes, i.e., genes present in 95% of the genomes, were identified with Roary version 3.13.0 [10], and a maximum-likelihood tree was calculated from the concatenated alignment of core genes (*n*=899) using the GTR+G model with 1,000 bootstrap replicates using RAxML version 0.9.0 [11] (Fig. 1).

Phylogenetic tree of L. reuteri displaying 14 numbered lineages, each comprising multiple strains. Colour-coding indicates organism origins from different hosts. Each bacterial strain shows subspecies classification and GenBank accession number.

The core-genome analysis identified 14 phylogenetic lineages within this species (Fig. 1). The topology of the phylogenetic tree is highly consistent with past reports on the phylogeny of lineages in *L. reuteri* that are based on core-genome phylogeny [4,5, 8] and with a phylogenetic tree that was inferred based on the GTR+F+I+R10 model using IQ-TREE (Fig. S1, available in the online Supplementary Material) [12]. Six of these lineages (lineages I–VI) correspond to established subspecies [5], while four lineages represented by isolates (lineages VII–X) [4] and four lineages not represented by isolates (lineages XI – XIV) do not cluster with the existing six *L. reuteri* subspecies [8].

To further assess the genomic similarities among strains, pairwise ANI and digital DNA–DNA hybridization (dDDH) values between them were calculated using OrthoANI [13] (Table 2) and the Genome-To-Genome Distance Calculator (GGDC) with blast algorithm [14] (Table 3) . The relatedness of strains of *L. reuteri* was compared to other subspecies of *L. reuteri* and to *Limosilactobacillus agrestis* and *Limosilactobacillus balticus*, the two species that are most closely related to *L. reuteri* [5,8]. ANI values within each of the lineages ranged from 96.8 to 99.7%, and dDDH values ranged from 72.6 to 99.0%. The two strains of lineage X (LR77^T^ and LR80) exhibited ANI values of 93.2–94.5% and dDDH values of 51.9–58.1% when compared to all other lineages including the type strain of *L. reuteri* DSM 20016^T^. The ANI and dDDH values of the remaining three lineages (VII, VIII and IX) relative to the type strain *L. reuteri* subsp. *reuteri* DSM 20016^T^ ranged from 95.6 to 96.6% and 65.0 to 72.8%, respectively (Table 2 and Table 3). When considering the thresholds for species delineation (ANI ≥95.5%, dDDH ≥70.0%) [15,17] and accounting for errors in genomic similarity calculations, the low ANI (< 95%) and dDDH (< 70%) values for lineage X support its classification as a novel species. The genomic distinctiveness of lineages VII, VIII and IX relative to existing *L. reuteri* subspecies supports their classification as new subspecies.

**Table 2.** ANI values (%) between the three novel *L. reuteri* subspecies, one newly proposed species, other type strains of identified subspecies of *L. reuteri* and the closely related species *L. agrestis* and *L. balticus*

Strain	*L. secundus* sp. nov.		*L. reuteri* subsp. *pararodentium* subsp. nov.		*L. reuteri* subsp. *peregrinus* subsp. nov.		*L. reuteri* subsp. *simiae* subsp. nov.	
	LR77^T^	LR80	LR92	mlc3^T^	LR51^T^	LR88	LR66^T^	LR52
***L. secundus sp. nov.***								
LR77^T^	—							
LR80	99.7	—						
***L. reuteri* subsp. *pararodentium* subsp. nov**.								
LR92	93.3	93.2	—					
mlc3^T^	93.3	93.3	98.9	—				
***L. reuteri* subsp. *peregrinus* subsp. nov**.								
LR51^T^	94.0	93.9	95.3	95.3	—			
LR88	93.9	93.8	95.3	95.4	97.6	—		
***L. reuteri* subsp*. simiae* subsp. nov**.								
LR66^T^	94.4	94.3	94.6	94.6	96.0	95.8	—	
LR52	94.2	94.0	94.3	94.4	95.4	95.5	96.8	—
***L. reuteri* subsp. *porcinus***								
3c6^T^	94.4	94.5	94.7	94.6	95.8	95.7	96.1	96.2
***L. reuteri* subsp. *kinnaridis***								
AP3^T^	93.8	93.9	94.5	94.1	95.3	95.1	96.3	96.7
***L. reuteri* subsp. *suis***								
ATCC 53608^T^	93.7	93.5	95.8	95.4	96.5	95.9	95.5	95.1
***L. reuteri* subsp. *murium***								
lpuph1^T^	94.2	94.2	95.0	94.8	95.8	96.0	95.9	95.8
***L. reuteri* subsp. *rodentium***								
10023^T^	93.7	93.6	96.1	96.1	95.9	95.8	94.9	94.8
***L. reuteri* subsp. *reuteri***								
DSM 20016^T^	94.4	94.5	95.6	95.7	96.6	96.6	96.1	95.8
***L. balticus***								
BG-AF3-A^T^	92.4	92.3	94.6	94.3	93.4	93.1	93.4	93.2
***L. agrestis***								
WF-MT5-A^T^	90.3	90.3	90.8	90.8	90.6	90.5	90.6	90.5

**Table 3.** dDDH values (%) between the three novel *L. reuteri* subspecies, one newly proposed species, other type strains of identified subspecies of *L. reuteri* and the closely related species *L. agrestis* and *L. balticus*

Strain	*L. secundus* sp. nov.		*L. reuteri* subsp. *pararodentium* subsp. nov.		*L. reuteri* subsp. *peregrinus* subsp. nov.		*L. reuteri* subsp. *simiae* subsp. nov.	
	LR77^T^	LR80	LR92	mlc3^T^	LR51^T^	LR88	LR66^T^	LR52
***L. secundus* sp*. nov.***								
LR77^T^	—							
LR80	99.0	—						
***L. reuteri* subsp*. pararodentium* subsp. nov**.								
LR92	52.5	52.0	—					
mlc3^T^	52.2	51.9	91.6	—				
***L. reuteri* subsp*. peregrinus* subsp. nov**.								
LR51^T^	55.5	55.2	63.2	63.3	—			
LR88	55.5	55.4	63.2	63.7	79.1	—		
***L. reuteri* subsp*. simiae* subsp*.* nov**.								
LR66^T^	57.5	57.2	58.9	58.6	67.2	65.8	—	
LR52	57.3	56.9	58.5	58.3	63.9	63.6	72.6	—
***L. reuteri* subsp*. porcinus***								
3c6^T^	57.9	58.1	59.8	59.3	66.1	65.6	69.5	69.3
***L. reuteri* subsp*. kinnaridis***								
AP3^T^	55.5	55.5	59.5	56.8	64.2	61.3	69.6	73.8
***L. reuteri* subsp*. suis***								
ATCC 53608^T^	54.2	54.1	67.2	64.6	71.3	67.1	64.2	62.2
***L. reuteri* subsp*. murium***								
lpuph1^T^	56.9	56.8	60.3	59.9	66.2	68.5	66.1	65.9
***L. reuteri* subsp*. rodentium***								
100-23^T^	54.0	53.8	68.8	69.1	66.2	65.8	60.4	60.3
***L. reuteri* subsp*. reuteri***								
DSM 20016^T^	58.1	57.9	65.0	65.7	72.5	72.8	68.1	66.7
***L. balticus***								
BG-AF3-A^T^	48.7	48.6	59.0	58.3	52.5	51.0	52.7	52.0
***L. agrestis***								
WF-MT5-A^T^	42.0	41.7	43.0	42.6	42.8	42.8	43.0	42.7

## Physiology

Carbohydrate fermentation profiles (Table 4) of *L. reuteri* lineage VII, VIII, IX and X were analysed using the API 50 CH system and compared to literature data for the established subspecies [5,18]. All strains of these four lineages fermented l-arabinose, d-ribose, d-galactose, d-glucose, maltose, lactose, melibiose, sucrose and raffinose. The utilization of d-xylose, d-fructose, starch and potassium gluconate was variable. *L. reuteri* subsp. *pararodentium* did not ferment d-fructose, starch or potassium gluconate but fermented d-xylose. *L. reuteri* subsp. *peregrinus* fermented potassium gluconate, but its ability to ferment d-xylose varied between strains: specifically, strain LR51^T^ was capable of d-xylose utilization, while strain LR88 was not. *L. reuteri* subsp. *simiae* strain LR52 fermented fructose but not starch, while the opposite is true for strain LR66^T^. *L. secundus* utilized potassium gluconate but not d-xylose or starch. None of the strains fermented glycerol, erythritol, d-arabinose, l-xylose, d-adonitol, methyl β-xylopyranoside, d-mannose, l-sorbose, l-rhamnose, dulcitol, inositol, d-mannitol, d-sorbitol, methyl α-mannopyranoside, methyl α-d-glucopyranoside, N-acetylglucosamine, amygdalin, arbutin, aesculin, salicin, cellobiose, trehalose, inulin, melezitose, glycogen, xylitol, gentiobiose, turanose, d-lyxose, d-tagatose, d-fucose, l-fucose, l-arabitol, d-arabitol, potassium 2-ketogluconate, or potassium 5-ketogluconate. The carbohydrate fermentation patterns of *L. reuteri* lineage VII to X thus generally match the pattern of other strains of *L. reuteri* [5,18].

**Table 4.** Carbohydrate metabolism of *L. secundus* sp. nov.*, L. reuteri* subsp. *pararodentium* subsp. nov., *L. reuteri* subsp. *peregrinus* subsp. nov. and *L. reuteri* subsp. *simiae* subsp. nov. profiled by API 50 CH/CHL system Grey shading indicates the sugar is utilized, and white shading indicates sugars are not utilized.

Substrate^*^	*L.* *secundus* sp. nov.		*L. reuteri* subsp. *kinnaridis*	*L. reuteri* subsp. *porcinus*	*L.* *reuteri* subsp. *murium*	*L.* *reuteri* subsp. *reuteri*	*L.* *reuteri* subsp. *suis*	*L.* *reuteri* subsp. *rodentium*	*L.* *reuteri* subsp. *pararodentium* subsp. nov.		*L.* *reuteri* subsp. *peregrinus* subsp. nov.		*L.* *reuteri* subsp. *simiae* subsp. nov.	
	LR77^T^	LR80	AP3^T†^	3 c6^T†^	lpuph1^T†^	DSM 20016^T^	ATCC 53608^T†^	100-23^T†^	LR92	mlc3^T^	LR51^T^	LR88	LR66^T^	LR52
l-Arabinose														
d-Ribose														
d-Xylose														
Galactose														
Glucose														
Fructose														
Maltose														
Sucrose														
Lactose														
Melibiose														
Raffinose														
Starch														
K-Gluconate														

* None of the strains produced acid from glycerol, erythritol, d-arabinose, l-xylose, d-adonitol, methyl *β*-xylopyranoside, d-mannose, l-sorbose, l-rhamnose, dulcitol, inositol, d-mannitol, d-sorbitol, methyl α-mannopyranoside, methyl α-glucopyranoside, *N*-acetylglucosamine, amygdalin, arbutin, aesculin, salicin, cellobiose, trehalose, inulin, melezitose, glycogen, xylitol, gentiobiose, turanose, d-lyxose, d-tagatose, d-fucose, l-fucose, L-arabitol, d-arabitol, potassium 2-ketogluconate and potassium 5-ketogluconate.

† API 50 CH test data of *L. reuteri* subsp. *kinnaridis, L. reuteri* subsp. *porcinus*, *L. reuteri* subsp. *murium*, *L. reuteri* subsp. *reuteri*, *L. reuteri* subsp. *suis* and *L. reuteri* subsp. *rodentium* was retrieved from [5,18].

## Discussion

Strains of *L. reuteri* and closely related species adapted to a series of vertebrate hosts. The ancestral hosts of *L. reuteri* are rodents (*Rodentiae*), and some subspecies appeared to specialize to the family *Muridae* [4]. Birds (class *Avium*) are a second host of *L. reuteri* subsp. *kinnaridis* for which host specialization has been documented experimentally [6]. The presence of *L. reuteri* in the intestine of humans was suggested to result from zoonotic transmission, or from consumption of *L. reuteri* with fermented foods [4]. The lineages of *L. reuteri* analysed herein all colonize mice and share the genomic traits of rodent-adapted lineages [4]. All isolates of *L. reuteri* lineage IX, however, were obtained from captive monkeys, which may reflect that domestication and captivity impact gut microbiota [19]. Our study, along with others [8], also identified four novel lineages of *L. reuteri* that are not represented by isolates. These novel lineages indicate that the diversity of *L. reuteri* and other gut commensals is far from being represented by current isolates or genomes, in part because wild animals remain under-represented as an isolation source. A value of 70% dDDH, corresponding to ANI values of about 95–95.5%, is widely accepted as the threshold for delineation of novel bacterial species, but a corresponding threshold value for delineation of bacterial subspecies has not been established [20,21]. For *Lactobacillaceae*, inter-subspecies dDDH and ANI values range from less than 70 and 96%, respectively, to more than 80 and 97%, respectively [22]. Subspecies of *L. reuteri* were previously described based on the phylogenetic position, the gene content of the genomes and lineage-specific host adaptation. These analyses also described four lineages that are not assigned to validly published subspecies [4,5].

### Proposal of one novel species and three novel subspecies within *L. reuteri*

According to the core-genome-based phylogenetic analyses, carbohydrate fermentation patterns and experimental evidence of biofilm formation in the forestomach of germ-free mice between the phylogenetic lineages [4], we propose that the four novel distinct phylogenetic lineages of *L. reuteri* reported previously and in the present study represent one novel species and three novel subspecies. We propose the following names: *Limosilactobacillus secundus* sp. nov. (type strain LR77^T^=DSM 113335^T^=LMG 32469^T^), *L. reuteri* subsp. *pararodentium* subsp. nov. (type strain mlc3^T^=DSM 113337^T^=LMG 32470^T^), *L. reuteri* subsp. *peregrinus* subsp. nov. (type strain LR51^T^=DSM 113336^T^=LMG 32467^T^) and *L. reuteri* subsp. *simiae* subsp. nov. (type strain LR66^T^=DSM 113334^T^ = LMG 32468^T^).

## Description of *Limosilactobacillus secundus* sp. nov.

*Limosilactobacillus secundus* sp. nov. (se.cun'dus. L. masc. adj. *secundus*, second, referring to the initial working name of the subspecies as ‘second species’).

Strains of *L. secundus* in this study were isolated from the striped field mouse (*A. agrarius*) and share ANI values of more than 99.7% with each other, 94.4–94.5% to the type strain of *L. reuteri* subsp. *reuteri* DSM200016 and 92.4% or less to other species of the genus *Limosilactobacillus* (Table 2) . Acid is produced from l-arabinose, d-ribose, d-galactose, d-glucose, maltose, lactose, melibiose, sucrose, raffinose and potassium gluconate but not from d-fructose, d-mannose, methyl *α*-d-glucopyranoside, aesculin, glycerol, erythritol, d-arabinose, d-xylose, l-xylose, d-adonitol, methyl *β*-d-xylopyranoside, l-sorbose, l-rhamnose, dulcitol, inositol, d-mannitol, d-sorbitol, methyl *α*-d-mannopyranoside, *N*-acetylglucosamine, amygdalin, arbutin, salicin, cellobiose, gentiobiose, trehalose, inulin, melezitose, starch, glycogen, xylitol, turanose, d-lyxose, d-tagatose, d-fucose, l-fucose, d-arabitol, l-arabitol, potassium 2-ketogluconate or potassium 5-ketogluconate. The origin of strains (Table S1), analysis of the gene content of the subspecies [4] and determination of biofilm formation in mice [4] document that strains of this subspecies colonize rodents. The type strain, LR77^T^ (=DSM 113335^T^, =LMG 32469^T^), was isolated from a striped field mouse (*A. agrarius*). The GenBank accession numbers for the genome sequence and the 16S rRNA gene sequence of the type strain are GCA_020784695.1 and PV954769, respectively. The genome has a size of 2.01 Mbp and a G+C content of 38.4 mol%.

## Description of *Limosilactobacillus reuteri* subsp. *pararodentium* subsp. nov.

*Limosilactobacillus reuteri* subsp. *pararodentium* (pa.ra.ro.den′ti.um. Gr. prep. *para*, resembling; N.L. *rodentium*, a subspecies epithet, N.L. gen. pl. n. *pararodentium*, resembling *L. reuteri* subsp. *rodentium*).

Strains of *L. reuteri* subsp. *pararodentium* were previously assigned to the subspecies *L. reuteri* subsp. *rodentium,* but analysis of strains on a larger scale, as performed in this study, demonstrates that *L. reuteri* subsp. *pararodentium* forms a monophyletic cluster (Fig. 1). Strains of this subspecies share more than 98.9% intra-subspecies ANI but less than 96.1% ANI to strains of other subspecies of *L. reuteri* and less than 94.6% ANI to other species of the genus *Limosilactobacillus* (Table 2). Strains of *L. reuteri* subsp. *pararodentium* were predominantly isolated from *Muridae* [4]. Acid is produced from l-arabinose, d-ribose, d-xylose, d-galactose, d-glucose, maltose, lactose, melibiose, sucrose and raffinose but not from d-fructose, d-mannose, methyl *α*-d-glucopyranoside, aesculin, potassium gluconate, glycerol, erythritol, d-arabinose, l-xylose, d-adonitol, methyl *β*-d-xylopyranoside, l-sorbose, l-rhamnose, dulcitol, inositol, d-mannitol, d-sorbitol, methyl *α*-d-mannopyranoside, *N*-acetylglucosamine, amygdalin, arbutin, salicin, cellobiose, gentiobiose, trehalose, inulin, melezitose, starch, glycogen, xylitol, turanose, d-lyxose, d-tagatose, d-fucose, l-fucose, d-arabitol, l-arabitol, potassium 2-ketogluconate or potassium 5-ketogluconate. The origin of strains of the subspecies (Table S1), the gene content of strains [4], literature data on the colonization of mice [4,6] and determination of biofilm formation in mice [23] demonstrate that strains of this subspecies can stably colonize rodents. The type strain, mlc3^T^ (=DSM 113337^T^, LMG 32470^T^), was isolated from a mouse (*M. musculus*). The GenBank accession numbers for the genome sequence and the 16S rRNA gene sequences of the type strain are GCA_000179435.1 and PV954770, respectively. The genome has a size of 2.02 Mbp and a G+C content of 38.5 mol%.

## Description of *Limosilactobacillus reuteri* subsp. *peregrinus* subsp. nov.

*Limosilactobacillus reuteri* subsp. *peregrinus* [pe.re.gri.nus L. masc. n. *peregrinus*, pilgrim, wanderer, referring to the ability of strains of the subspecies to (temporarily) persist in phylogenetically diverse hosts].

Strains of *L. reuteri* subsp. *peregrinus* form a monophyletic cluster (Fig. 1) and share more than 97.6% intra-subspecies ANI, less than 96.6% ANI to strains of other subspecies of *L. reuteri* and less than 93.4% ANI to other species of the genus *Limosilactobacillus* (Table 2). *L. reuteri* subsp. *peregrinus* were isolated from humans, primates and rodents [4]. Acid is produced from l-arabinose, d-ribose, d-galactose, d-glucose, maltose, lactose, melibiose, sucrose, raffinose and potassium gluconate. Acid-producing pattern is variable for d-xylose, and acid is not produced from d-fructose, d-mannose, methyl *α*-d-glucopyranoside, aesculin, glycerol, erythritol, d-arabinose, l-xylose, d-adonitol, methyl *β*-d-xylopyranoside, l-sorbose, l-rhamnose, dulcitol, inositol, d-mannitol, d-sorbitol, methyl *α*-d-mannopyranoside, *N*-acetylglucosamine, amygdalin, arbutin, salicin, cellobiose, gentiobiose, trehalose, inulin, melezitose, starch, glycogen, xylitol, turanose, d-lyxose, d-tagatose, d-fucose, l-fucose, d-arabitol, l-arabitol, potassium 2-ketogluconate or potassium 5-ketogluconate. The origin of strains of the subspecies (Table S1), analysis of the gene content of strains [4] and determination of biofilm formation in mice [23] demonstrate that strains of this subspecies colonize rodents but also (temporarily) persist in other hosts. The type strain, LR51^T^ (=DSM 113336^T^, LMG 32467^T^), was isolated from a black howler monkey (*A. caraya*). The GenBank accession numbers for the genome sequence and the 16S rRNA gene sequences of the type strain are GCA_020785115.1 and PV954765, respectively. The genome has a size of 2.10 Mbp and a G+C content of 38.6 mol%.

## Description of *Limosilactobacillus reuteri* subsp. *simiae* subsp. nov.

*Limosilactobacillus reuteri* subsp. *simiae* (si’mi.ae L. gen. n. *simiae*, of a monkey, referring to the origin of all known strains of the subspecies).

Strains of *L. reuteri* subsp. *simiae* form a monophyletic cluster (Fig. 1). They share more than 96.8% intra-subspecies ANI, less than 96.7% ANI to strains of other subspecies of *L. reuteri* and less than 93.4% ANI to other species of the genus *Limosilactobacillus* (Table 2). *L. reuteri* subsp. *simiae* were isolated from captive black howler monkeys and patas monkeys [4]. Acid is produced from l-arabinose, d-ribose, d-galactose, d-glucose, maltose, lactose, melibiose, sucrose, raffinose and potassium gluconate; acid production from d-fructose and starch is variable and acid is not produced from d-mannose, methyl α-d-glucopyranoside, aesculin, glycerol, erythritol, d-arabinose, d-xylose, l-xylose, d-adonitol, methyl *β*-d-xylopyranoside, l-sorbose, l-rhamnose, dulcitol, inositol, d-mannitol, d-sorbitol, methyl *α*-d-mannopyranoside, *N*-acetylglucosamine, amygdalin, arbutin, salicin, cellobiose, gentiobiose, trehalose, inulin, melezitose, glycogen, xylitol, turanose, d-lyxose, d-tagatose, d-fucose, l-fucose, d-arabitol, l-arabitol, potassium 2-ketogluconate or potassium 5-ketogluconate. The origin of strains of the subspecies (Table S1), analysis of the gene content of strains [4] and determination of biofilm formation in mice [23] demonstrate that strains of this subspecies colonize rodents but also (temporarily) persist in primates. The type strain, LR66^T^ (=DSM 113334^T^=LMG 32468^T^), was isolated from a patas monkey (*E. patas*). The GenBank accession numbers for the genome sequence and the 16S rRNA gene sequences of the type strain are GCA_020784875.1 and PV954768, respectively. The genome has a size of 2.16 Mbp and a G+C content of 38.5 mol%.

## Supplementary material

10.1099/ijsem.0.007099 Supplementary Material 1.

10.1099/ijsem.0.007099 Uncited Supplementary Material 2.

## Abbreviations

ANI: average nucleotide identity
dDDH: digital DNA–DNA hybridization
