# Supplementary material for: Proposal of Limosilactobacillus secundus sp. nov., Limosilactobacillus reuteri subsp. pararodentium subsp. nov., Limosilactobacillus reuteri subsp. peregrinus subsp. nov. and Limosilactobacillus reuteri subsp. simiae subsp. nov., isolated from the gastrointestinal tract of vertebrate hosts
Source: Int J Syst Evol Microbiol. 2026 Mar 6;76(3):007099. doi: 10.1099/ijsem.0.007099 (PMC12967093; doi:10.1099/ijsem.0.007099)
Supplement: Supplementary Material 1. [file ijsem-76-07099-s001.pdf]

Online supplementary material to

Proposal of *Limosilactobacillus secundus* sp. nov., *Limosilactobacillus reuteri* subsp. *pararodentium* subsp. nov., *Limosilactobacillus reuteri* subsp. *peregrinus* subsp. nov., and *Limosilactobacillus reuteri* subsp. *simiae* subsp. nov., isolated from the gastrointestinal tract of vertebrate hosts

Xinyu Guo, Yi Yang, Justina Su Zhang, Peipei Zhang, Jens Walter, Michael G. Gänzle, Fuyong Li

Figure S1. A maximum-likelihood phylogenetic tree was constructed using core genes identified from whole-genome sequences. The tree was inferred based on the GTR+F+I+R10 model with 1000 bootstrap replicates and all bootstrap values are above 80%.

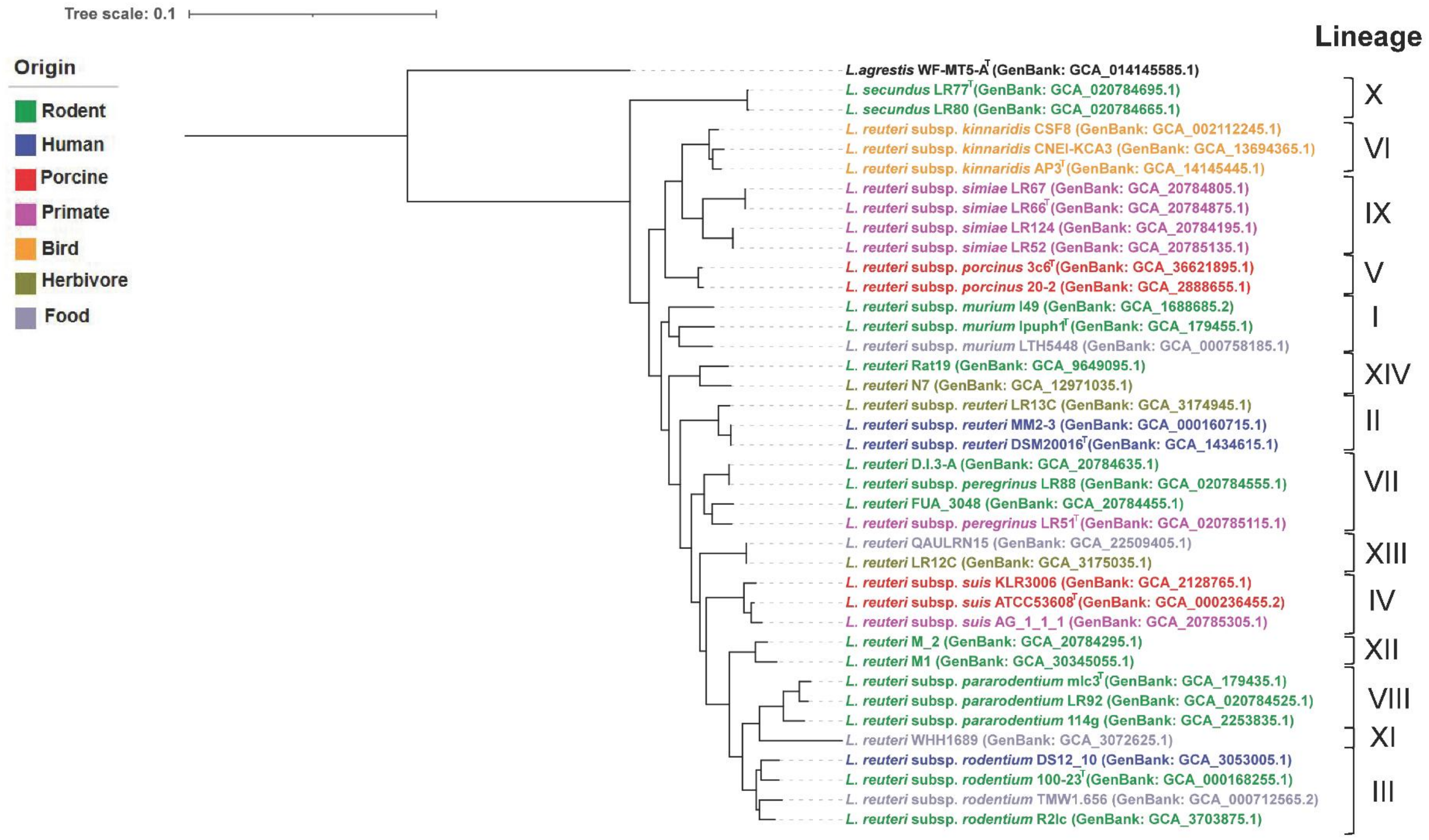

Figure S1. A maximum-likelihood phylogenetic tree was constructed using core genes identified from whole-genome sequences. The tree was inferred based on the GTR+F+I+R10 model with 1000 bootstrap replicates and all bootstrap values are above 80%. Genome sequences for the type strains were obtained from GenBank database. The 14 phylogenetic lineages of *Limosilactobacillus reuteri* are numbered with roman numerals to the right. The phylogenetic tree was visualized using iTOL [24].
